# Dr. Solomon Bueno de Mesquita

**Published:** 1909-04-03

**Authors:** 


Dr. Solomon Bueno de Mesquita died at Margate at the age of forty-seven. He became a member of the Royal College of Surgeons in 1887, a Licentiate of the Royal College of Physicians in 1888, while he obtained the Bachelor of Science and Doctor of Medicine degrees of the University of London five years later. Dr. Mesquita was visiting physician to the South Tottenham Home for Incurables, and had been house surgeon to the Miller Hospital, Greenwich, and the Croydon General Hospital.

